# Antihyperuricemic Effects of *Cornus officinalis* Extract via URAT1 Regulation and Renoprotective Mechanisms

**DOI:** 10.3390/ijms26209980

**Published:** 2025-10-14

**Authors:** Yoon-Young Sung, Dong-Seon Kim, Seung-Hyung Kim, Heung Joo Yuk

**Affiliations:** 1 KM Science Research Division, Korea Institute of Oriental Medicine, 1672 Yuseongdae-ro, Yuseong-gu, Daejeon 34054, Republic of Korea; yysung@kiom.re.kr (Y.-Y.S.); dskim@kiom.re.kr (D.-S.K.); 2 DJU Industry-University Cooperation Foundation, Daejeon University, 62 Daehak-ro, Dong-gu, Daejeon 34520, Republic of Korea; sksh518@dju.kr

**Keywords:** gout, kidney disease, *Cornus officinalis*, URAT1 regulation, uric acid excretion

## Abstract

Hyperuricemia, characterized by elevated serum uric acid levels, is a major risk factor for gout and kidney disease. This study evaluated the antihyperuricemic effects of *Cornus officinalis* extract (COE) using urate transporter 1 (URAT1)-expressing oocytes and a hyperuricemia rat model. COE effectively inhibited uric acid absorption by modulating URAT1, with an IC_50_ value of 3.24 µg/mL. In the hyperuricemia model, COE administration (100 and 200 mg/kg) significantly reduced serum uric acid levels and increased urinary uric acid excretion. The primary constituents of COE, morroniside (MO) and loganin (LO) exerted similar effects, with MO exhibiting potent inhibition of uric acid absorption even at low concentrations. Kidney tissue analysis revealed a reduction in blood urea nitrogen (BUN) levels, indicating improved renal function. Liver function parameters (ALT, AST, and LDH) remained unchanged, suggesting an absence of hepatotoxicity. Ultra-high-performance liquid chromatography with charged aerosol detection (UHPLC-CAD) analysis identified MO (17.8 mg/g), LO (9.8 mg/g), and cornin (1.4 mg/g) as the principal components of COE. These findings suggest that COE enhances uric acid excretion via URAT1 regulation and exerts renoprotective effects, highlighting its potential as an antihyperuricemic agent. Furthermore, MO and LO were identified as the primary active constituents, and COE appears to be a promising therapeutic candidate with a favorable safety profile.

## 1. Introduction

Hyperuricemia is a metabolic disorder characterized by abnormally elevated serum uric acid levels, which can lead to various complications, including gout, chronic kidney disease, and cardiovascular disease [1]. The primary cause of hyperuricemia is an imbalance between uric acid production and excretion, with renal uric acid reabsorption playing a crucial role in maintaining uric acid homeostasis [2]. Among the several urate transporters in the kidney, urate transporter 1 (URAT1) is a key regulator of urate reabsorption in the proximal tubule. Inhibiting URAT1 to enhance urate excretion is considered an effective therapeutic strategy for hyperuricemia [3]. Currently, representative URAT1 inhibitors, such as benzbromarone and lesinurad, are used to lower serum uric acid levels by promoting uric acid excretion [4]. However, these synthetic agents are associated with adverse effects, including hepatotoxicity and nephrotoxicity, which limit their long-term use [5]. Consequently, natural product-based URAT1 inhibitors with improved safety profiles are gaining increasing attention as potential therapeutic alternatives.

*Cornus officinalis*, a member of the Cornaceae family, has been widely used as a traditional herbal medicine in Korea, China, and Japan. Studies on the bioactive properties of *C. officinalis* have reported anticancer, antidiabetic, anti-inflammatory, antibacterial, and antioxidant effects. The major phytochemical constituents of *C. officinalis* include phenolic acids, triterpenes, iridoids, and tannins, with morroniside (MO) and loganin (LO) identified as the principal iridoids [6]. Previous studies suggest that MO and LO may exert nephroprotective effects through their antioxidant and anti-inflammatory activities [7,8]. Moreover, *C. officinalis* extracts have been reported to exert hypouricemic effects in hyperuricemia-induced cell and mouse models [9], further supporting its potential role in uric acid regulation. However, their direct effects on uric acid metabolism, particularly in relation to URAT1 regulation, remain unexplored. Furthermore, the effects of *C. officinalis* extract (COE) and its active components on URAT1-mediated uric acid transport have not been investigated. Therefore, this study aimed to evaluate whether COE and its major constituents (MO and LO) promote uric acid excretion by modulating URAT1 and to assess their protective effects against hyperuricemia-induced renal dysfunction and histopathological damage. By elucidating these effects, we sought to explore their potential as natural product-based drug candidates for hyperuricemia treatment. To evaluate the in vivo antihyperuricemic efficacy of COE and its constituents, hyperuricemia was induced in rats by administering potassium oxonate (PO), a uricase inhibitor. This PO-induced model is widely used to mimic human hyperuricemia, as it elevates serum uric acid levels by inhibiting uric acid degradation and subsequently induces renal impairment. This well-established animal model enables the reliable assessment of therapeutic interventions targeting hyperuricemia [10].

Additionally, an analytical study using ultra-high-performance liquid chromatography (UHPLC) coupled with a charged aerosol detector (CAD) was conducted to quantitatively analyze the key components of COE. CAD enables accurate detection of compounds lacking UV absorption, facilitating precise identification of the main constituents of the extract. The quantitative analysis of these bioactive components is expected to provide baseline data for future bioactivity studies on COE and contribute to the standardization of raw materials for drug development.

## 2. Results

### 2.1. Chemical Profiling and Quantitative Analysis by UHPLC-CAD

**Figure 1:**
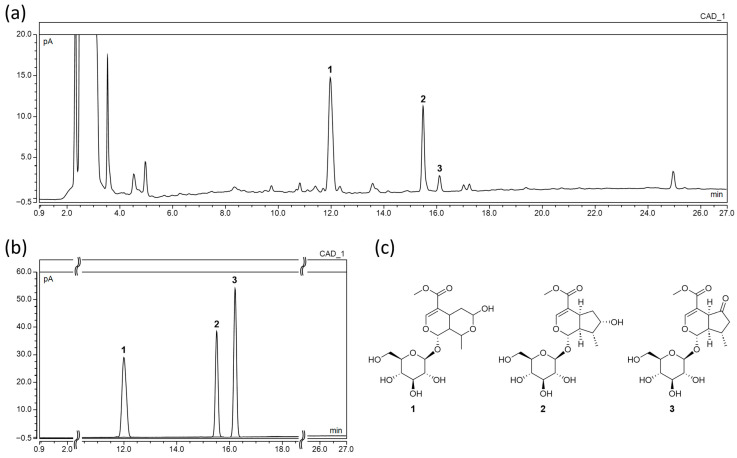
Representative UHPLC-CAD chromatogram of (**a**) *Cornus officinalis* extract (COE); (**b**) overlay chromatogram of standard compounds morroniside (MO, **1**), loganin (LO, **2**), and cornin (CO, **3**); and (**c**) chemical structures of compounds **1**–**3**.

Chromatographic analysis of MO, LO, and cornin (CO) in both standard solutions and COE confirmed their separation without interference from other substances (Figure 1). The retention times (RTs) of the standard and COE samples were consistent: 11.97 min for peak 1 (MO), 15.49 min for peak 2 (LO), and 16.10 min for peak 3 (CO). Linearity was verified by constructing calibration curves using standard solutions at six or more concentrations, each measured in triplicate. The correlation coefficients (r^2^) for all three compounds exceeded 0.999, indicating excellent linearity. Quantitative analysis of COE identified MO (17.8 mg/g) as the most abundant functional compound, followed by LO (9.8 mg/g) and CO (1.4 mg/g).

### 2.2. Effects of COE on In Vitro XOD Activity

**Figure 2:**
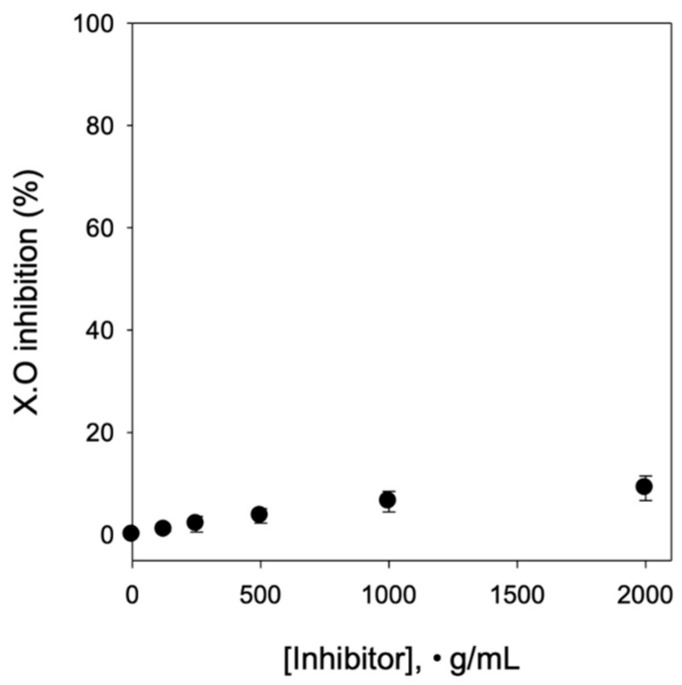
Effect of COE on in vitro xanthine oxidase (XOD) activity. *n* = 5 per group. COE, *Cornus officinalis* extract.

To evaluate the effect of COE on uric acid synthesis, in vitro XOD activity was measured. As shown in Figure 2, COE did not inhibit XOD activity, indicating that COE does not suppress uric acid synthesis.

### 2.3. Effects of COE, LO, MO, and CO on Urate Excretion in In Vitro URAT1-Expressing Oocytes

**Figure 3:**
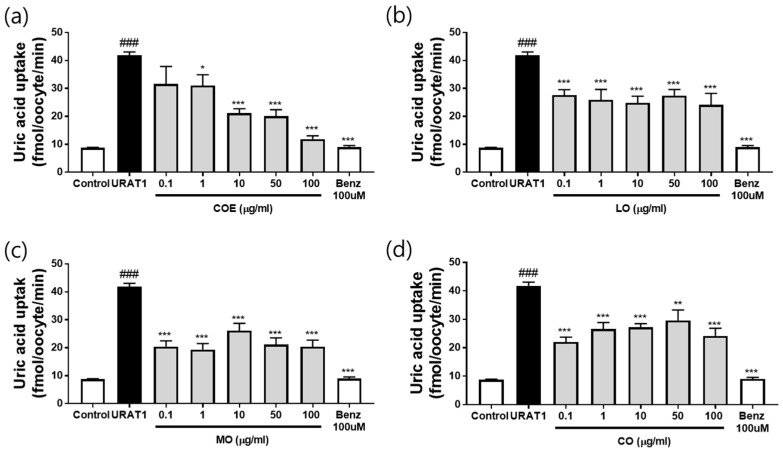
Effect of COE and its constituents (LO, MO, and CO) on uric acid uptake in in vitro human urate transporter 1 (hURAT1)-overexpressing oocytes. (**a**) Inhibitory effect of COE at 0.1–100 µg/mL; (**b**) Effect of LO; (**c**) Effect of MO; (**d**) Effect of CO. *n* = 5 per group. COE, *Cornus officinalis* extract; LO, loganin; MO, morroniside; CO, cornin; Benz, benzbromarone. Control, non-injected oocytes; URAT1, URAT1-injected oocytes + vehicle. ### *p* < 0.001 vs. the control group; * *p* < 0.05, ** *p* < 0.01, and *** *p* < 0.001 vs. the URAT1 group.

The effects of COE and its constituents (LO, MO, and CO) on urate excretion were assessed using an in vitro urate uptake system in hURAT1-overexpressing oocytes (Supplementary Materials Figure S1). COE treatment (0.1, 1, 10, 50, and 100 µg/mL) exhibited a potent, dose-dependent inhibitory effect on uric acid uptake (Figure 3a), with an IC_50_ value of 3.24 µg/mL. Among the individual components, LO inhibited uric acid uptake at 100 µg/mL, achieving >50% inhibition (predictive IC_50_ >100 µg/mL, Figure 3b). MO demonstrated strong inhibitory activity across the tested concentrations (0.1–100 µg/mL), reducing uric acid uptake by more than 64% (predictive IC_50_ <0.1 µg/mL, Figure 3c). MO strongly inhibited urate uptake even at 0.1 µg/mL (64% reduction), and the IC_50_ was predicted to be <0.1 µg/mL. Since concentrations below 0.1 µg/mL were not tested, further experiments are required to more precisely determine the IC_50_. In contrast, CO exhibited weak inhibition (0.1–100 µg/mL) with a predictive IC_50_ >100 µg/mL (Figure 3d). These results indicate that COE and its constituents promote urate excretion by inhibiting uric acid uptake, resembling the mechanism of the URAT1 inhibitor benzbromarone. No morphological toxicity was observed in oocytes at any tested concentration.

### 2.4. Effects of COE, LO, and MO on Serum and Urine Uric Acid Levels

**Figure 4:**
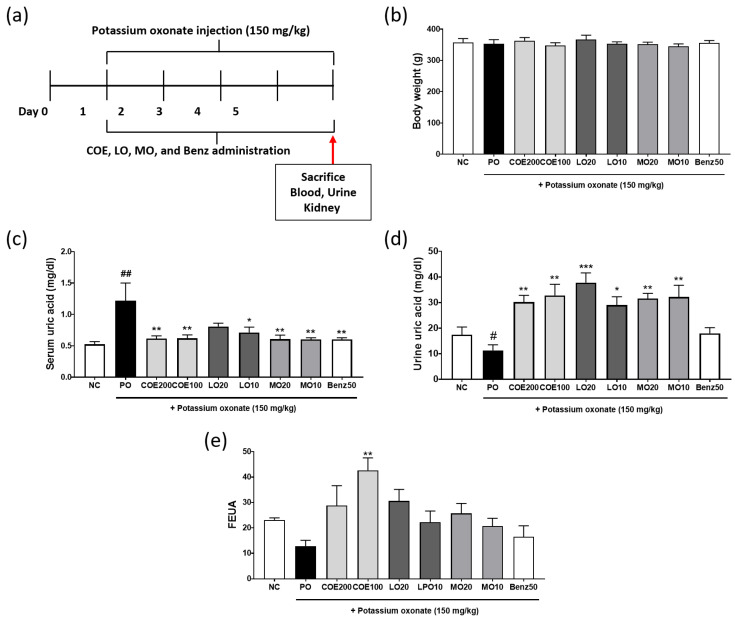
Effects of COE, LO, and MO on serum and urinary uric acid levels in rats with potassium oxonate (PO)-induced hyperuricemia. (**a**) Experimental design, (**b**) body weight, (**c**) serum uric acid levels, (**d**) urinary uric acid levels, and (**e**) FEUA. *n* = 5 per group. COE, *Cornus officinalis* extract; LO, loganin; MO, morroniside; Benz, benzbromarone. # *p* < 0.05 and ## *p* < 0.01 vs. the normal control group; * *p* < 0.05, ** *p* < 0.01, and *** *p* < 0.001 vs. the PO group.

The effects of COE, LO, and MO were evaluated in PO-induced hyperuricemic rats. The experimental design is illustrated in Figure 4a. No significant differences in body weight were observed among the groups (Figure 4b). PO injection for five days significantly [*p* = 0.0004, effect size (95% CI): 0.6938 (0.2551~1.132)] increased serum uric acid levels in the PO group compared with the normal control group (Figure 4c). Administration of COE [100 mg/kg; *p* = 0.0029, 0.5966 (0.1579~1.035) and 200 mg/kg; *p* = 0.0026, 0.6016: 0.1629~1.04)] and the positive control benzbromarone [*p* = 0.0021, 0.6138 (0.1751~1.052)] significantly reduced serum uric acid levels compared with the PO group. Similarly, LO 10 mg/kg [*p* = 0.0162, 0.5038 (0.06513~0.9425)] and MO (10 and 20 mg/kg) administration also led to a reduction in serum uric acid levels [*p* = 0.0021, 0.6138 (0.1751~1.052) and *p* = 0.0022, 0.6102 (0.1715~1.049)].

To assess the effect of COE on uric acid excretion, urine uric acid levels were measured. The PO group exhibited a significant decrease in urinary uric acid levels (*p* = 0.049), which were restored following administration of COE (100 and 200 mg/kg) [*p* = 0.0019, −21.47 (−36.07~−6.864) and *p* = 0.0071, −18.87 (−33.47~−4.264)], LO (10 and 20 mg/kg) [*p* = 0.0127, −17.67 (−32.27~−3.064) and *p* = 0.0002, −26.47 (−41.07~−11.866)] and MO (10 and 20 mg/kg) [*p* = 0.0026, −20.87 (−35.47~−6.264) and *p* = 0.0036, −20.27 (−34.87~−5.664)] (Figure 4d). COE (100 mg/kg) administration led to increase in FEUA levels [*p* = 0.0020, −29.97 (−50.45~−9.491)] (Figure 4e). These findings suggest that COE enhances renal urate excretion, thereby lowering serum uric acid levels in hyperuricemic rats.

### 2.5. Effects of COE, LO, and MO on Kidney Function

**Figure 5:**
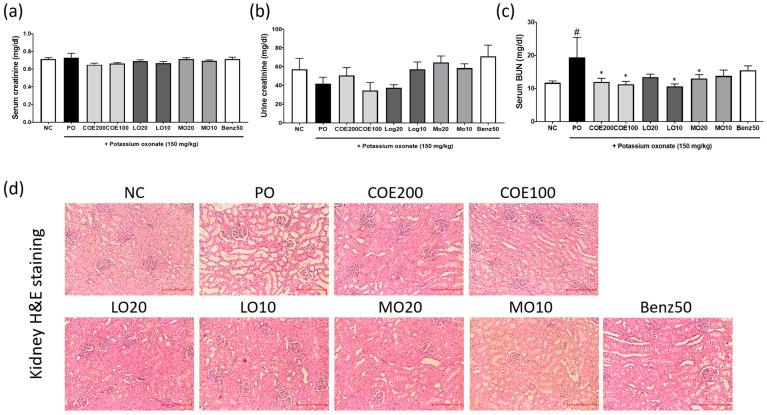
Effects of COE, LO, and MO on kidney function parameters in rats with potassium oxonate (PO)-induced hyperuricemia. (**a**) Serum creatinine, (**b**) urinary creatinine, (**c**) serum blood urea nitrogen (BUN), and (**d**) kidney tissue histopathology (H&E staining, original magnification 100×). *n* = 5 per group. COE, *Cornus officinalis* extract; LO, loganin; MO, morroniside; Benz, benzbromarone. # *p* < 0.05 vs. the normal control group; * *p* < 0.05 vs. the PO group.

Serum creatinine levels were measured to assess kidney function, as hyperuricemia is associated with renal impairment [2]. No significant differences in serum or urinary creatinine levels were observed among the groups (Figure 5a,b). However, serum BUN levels were elevated in the PO group [*p* = 0.0401, 7.683 (0.2619~15.1)], whereas administration of COE (100 and 200 mg/kg) [(*p* = 0.0192, 6.153 (1.057~15.25) and *p* = 0.0378, 7.413 (0.3171~14.51)], LO (10 mg/kg) [*p* = 0.0106, 8.773 (1.677~15.87)], and MO (20 mg/kg) [0.0499, 6.433 (−0.6629~13.53)] significantly reduced BUN levels (Figure 5c).

Histopathological analysis revealed mild renal tubular dilatation, swelling, vacuolar degeneration of tubular epithelial cells, and inflammatory cell infiltration in the kidneys of PO-injected hyperuricemic rats (Figure 5d). However, COE, its active components, and benzbromarone effectively ameliorated these renal histopathological changes. These results indicate that COE mitigates kidney dysfunction induced by PO treatment in hyperuricemic rats. In addition, Western blot analysis showed that COE administration decreased renal URAT1 protein expression in PO-induced rats (Supplementary Figure S2).

### 2.6. Effects of COE, LO, and MO on Liver Function

**Figure 6:**
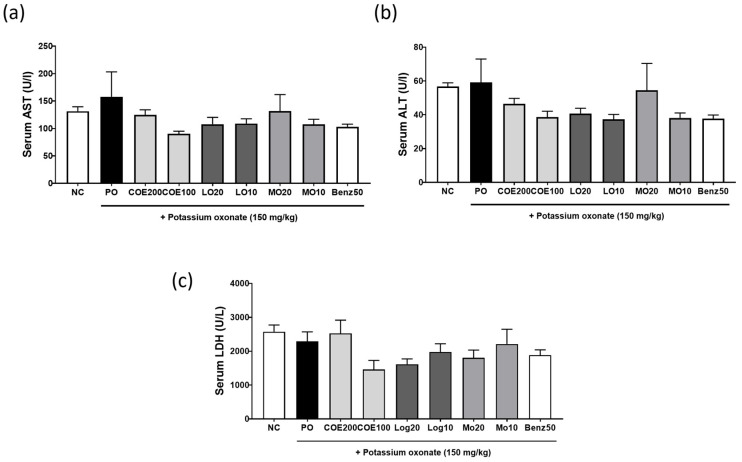
Effects of COE, LO, and MO on liver function parameters in rats with potassium oxonate (PO)-induced hyperuricemia. (**a**) Serum aspartate aminotransferase (AST), (**b**) alanine aminotransferase (ALT), and (**c**) lactate dehydrogenase (LDH) levels. *n* = 5 per group. COE, *Cornus officinalis* extract; LO, loganin; MO, morroniside; Benz, benzbromarone.

Elevated serum uric acid levels have been associated with hepatic damage [11]. To assess the effect of COE on liver function in hyperuricemic rats, serum levels of ALT, AST, and LDH were measured. As shown in Figure 6a–c, there were no significant differences in ALT, AST, or LDH levels among the groups.

## 3. Discussion

The present study successfully established a reliable analytical method for the quantification of key bioactive compounds in COE, including MO, LO, and CO. These findings not only demonstrate the suitability of the UHPLC-CAD method for accurately quantifying non-UV-absorbing constituents in complex herbal extracts, but also underscore the necessity of standardizing the extraction process for pharmaceutical development.

CAD detection was particularly advantageous, as it enables the detection of analytes that do not possess chromophores. This is especially relevant for morroniside, loganin, and cornin, which are polar iridoid glycosides with minimal UV absorption. Conventional UV detectors may yield low sensitivity for such compounds, whereas CAD provides consistent and sensitive detection based on aerosolized particle charge, making it ideal for accurate quantification of these pharmacologically active components.

Hyperuricemia results from excessive uric acid production, impaired uric acid excretion, or a combination of both processes, leading to gout [12,13]. XOD is the key rate-limiting enzyme responsible for the conversion of endogenous purines into uric acid [14]. XOD inhibitors, such as allopurinol, suppress uric acid synthesis, thereby reducing uric acid production and lowering serum uric acid levels [15,16]. In this study, the inhibitory effect of COE on XOD activity in vitro was assessed before evaluating its antihyperuricemic activity in animal models. Our results indicated that COE did not inhibit XOD activity, suggesting that COE does not modulate uric acid synthesis.

Approximately 90% of urate reabsorption occurs through renal urate transporter 1 (URAT1) [17]. URAT1 inhibitors, such as benzbromarone, are considered promising uricosuric agents for hyperuricemia treatment [18,19]. Therefore, in this study, the inhibitory effects of COE and its three primary constituents (LO, MO and CO), as identified by UHPLC-CAD analysis, upon URAT1-mediated urate uptake were investigated using URAT1-overexpressing oocytes. COE and its constituents effectively inhibited URAT1-mediated urate uptake, suggesting that COE reduces hyperuricemia-associated increases in uric acid levels by promoting uric acid excretion. Although these compounds exhibited URAT1 inhibitory activity, their efficacy in hyperuricemia has not been previously reported.

To further evaluate the antihyperuricemic effects of COE and its active compounds, their efficacy was confirmed in PO-induced hyperuricemic rats. COE, LO, and MO (particularly MO) significantly reduced serum uric acid levels and increased urinary uric acid excretion in PO-induced hyperuricemic rats without affecting body weight. These findings suggest that COE enhances renal uric acid excretion by regulating URAT1 activity, thereby alleviating hyperuricemia. Additionally, LO and MO were identified as the bioactive compounds responsible for this effect, as they effectively suppressed hyperuricemia in the animal model. While the impact of these compounds on hyperuricemia has not been previously reported, LO has been shown to mitigate hyperuricemia-induced gout. Specifically, oral administration of LO was reported to alleviate gout-associated inflammation in a monosodium urate crystal-induced acute foot gout model in mice [8].

Renal urate excretion is regulated by multiple transporters, including organic anion transporters (OAT1, OAT2, and OAT3), which facilitate urate secretion, and OAT4, OAT10, and glucose transporter 9 (GLUT9), along with URAT1, which mediate urate reabsorption [20,21]. From the preliminary experiments, COE at 200 mg/kg significantly lowered URAT1 protein levels in the kidney (Supplementary Figure S2), supporting the hypothesis that COE promotes urate excretion through URAT1 modulation. However, this study did not assess the effects of COE and its compounds on these urate transport systems in hyperuricemic rats. Therefore, further research is required to elucidate the precise mechanisms by which LO and MO regulate urate excretion in hyperuricemia.

Hyperuricemia is a known risk factor for kidney dysfunction [22]. In this study, kidney function was assessed by measuring serum and urinary levels of creatinine and BUN, alongside renal histopathological analysis using H&E staining. Serum and urinary creatinine levels did not differ significantly between groups. However, the elevated serum BUN levels induced by PO treatment were ameliorated following COE, LO, and MO administration. Histological analysis further revealed that renal damage was mitigated in COE-, LO-, and MO-treated rats. Additionally, serum levels of AST, ALT, and LDH, markers of liver function, remained unchanged across all groups. These findings suggest that COE, containing LO and MO as major active compounds, may improve hyperuricemia without causing adverse effects, supporting its potential therapeutic application in humans. Although MO and LO showed antihyperuricemic effects at 10–20 mg/kg, these doses are higher than the amounts present in COE (≈3.56 and 1.96 mg/kg, respectively, at 200 mg/kg COE). Thus, the antihyperuricemic activity of COE may involve synergistic or additive contributions from other constituents. Future studies will evaluate MO and LO in combination at COE-equivalent doses to clarify their roles in the extract’s overall efficacy.

## 4. Materials and Methods

### 4.1. COE Preparation and Compound Profiling

The fruit of *Cornus officinalis* used in this study was obtained from Omniherb (Daegu, Republic of Korea) (http://www.omniherb.com/) (accessed on 2 October 2025), a certified supplier that adheres to the standards of the Korean pharmacopeia. The fruit was sourced from Gurye-gun, Jeollanam-do, South Korea. The extract was prepared by refluxing 0.5 kg of dried berries in 6 L of distilled water for 4 h. The resulting solution was filtered, concentrated under reduced pressure at 50 °C, and lyophilized to obtain a powdered extract for chemical profiling and biological activity assessments. MO, LO, and CO were used as standard compounds for chemical profiling and were purchased from ChemFaces (Wuhan, Hubei, China). For quantitative analysis, calibration curves were established using six different concentrations (500, 200, 100, 50, 20, and 10 µg/mL), yielding high linearity (r^2^ > 0.999). All solvents used for extraction and chromatographic analysis were of HPLC grade (J.T. Baker, Phillipsburg, NJ, USA), and formic acid (Sigma-Aldrich, St. Louis, MO, USA) was used to adjust the pH of the mobile phase.

### 4.2. UHPLC-CAD Analysis of COE

Ultra-high-performance liquid chromatography (UHPLC) coupled with CAD was employed for the quantitative analysis of COE’s major bioactive components. The analysis was conducted using a Dionex Ultimate 3000 UHPLC system (Thermo Fisher Scientific, Bremen, Germany) with a Waters XBridge^®^ C18 column (4.6 mm × 250 mm, 5 µm). The mobile phase consisted of water with 0.1% formic acid (solvent A) and acetonitrile with 0.1% formic acid (solvent B), with a flow rate of 1 mL/min. The sample injection volume was 10 µL, and elution was performed under the following gradient conditions: The initial mobile phase ratio was 95% solvent A, maintained for 1 min, followed by a decrease to 75% A over 25 min. At 26 min, the gradient transitioned to 100% solvent B, which was maintained for 8 min for column washing. The mobile phase was then re-equilibrated by returning to 95% solvent A within 1 min (34–35 min) and maintaining this ratio until 40 min. The CAD (Corona Veo RS) was operated at a nitrogen gas pressure of 40 psi and an evaporation temperature of 50 °C. The detector response was set at 100 pA, and a high-noise filter was applied to improve accuracy. Data acquisition and quantitative calculations were performed using the Chromeleon data system software (version 7.2.8).

### 4.3. In Vitro XOD Activity Assay

The in vitro xanthine oxidase (XOD) inhibitory activity was evaluated using bovine milk XOD, following previously published methods [23]. Test samples were prepared by dissolving COE in dimethyl sulfoxide (DMSO) and diluting it to various concentrations (0–300 µg/mL). The reaction mixture (total volume: 200 µL) was prepared as follows: Pre-incubation: 125 µL of 100 mM sodium pyrophosphate buffer (HCl, pH 7.5), 30 µL of XOD enzyme (0.1 units), and 5 µL of test sample were incubated at 37 °C. Substrate Addition: The reaction was initiated by adding 40 µL of xanthine (0.5 mM). Absorbance Measurement: The mixture was incubated in a 96-well plate (300 µL capacity) at 37 °C, and absorbance was measured at 295 nm to assess XOD activity.

### 4.4. Urate Uptake in URAT1-Expressing Oocytes

The hURAT1-expressing *Xenopus* oocyte system was established as described previously [24]. Oocytes were preincubated in assay buffer containing Dulbecco’s phosphate-buffered saline (DPBS; Sigma-Aldrich, St. Louis, MO, USA) and ND96 solution (96 mM NaCl, 2 mM KCl, 1.8 mM CaCl_2_, 1 mM MgCl_2_, and 5 mM HEPES, pH 7.4) supplemented with 1 mM pyrazine carboxylic acid (Sigma-Aldrich) as trans-stimulator [25,26,27,28,29,30] at 37 °C. Following preincubation, oocytes were further incubated in a solution containing 50 µM [^14^C] uric acid (Moravek Biochemicals, Brea, CA, USA) with various concentrations of COE, MO, and LO (0.1, 1, 10, and 100 µg/mL) for 60 min at 37 °C. Uric acid uptake was terminated by adding ice-cold DPBS, followed by thorough washing with the same solution. Oocytes were then lysed in a solution containing 0.1 N NaOH and 10% sodium dodecyl sulfate, and radioactivity was quantified using liquid scintillation counting. Benzbromarone (TCI, Tokyo, Japan), a URAT1 inhibitor, was used as a reference compound.

### 4.5. Animal Model

Seven-week-old male Sprague Dawley rats were obtained from ORIENT BIO (Seongnam, Republic of Korea). The animals were housed in an air-conditioned room at 22 ± 2 °C with a relative humidity of 55% ± 15% under a 12-h light/dark cycle. The rats were provided with a standard laboratory diet and water ad libitum. All animal experiments were approved by the Institutional Animal Care and Use Committee of Daejeon University and conducted in accordance with the committee’s guidelines (Approval Code: DJUARB2024-022).

### 4.6. Induction of Hyperuricemia and COE, LO, and MO Administration

Hyperuricemia was induced by intraperitoneal injection of potassium oxonate (PO, 150 mg/kg; Sigma-Aldrich, USA), a uricase inhibitor, for 5 consecutive days. Benzbromarone (Sigma-Aldrich, USA) was used as a positive control. Before injection, PO was dissolved in 0.5% carboxymethyl cellulose (CMC, pH 5.0) containing 0.1 M sodium acetate.

To evaluate the antihyperuricemic effects of COE, LO, and MO, the rats were randomly divided into nine groups (*n* = 5 per group): (1) Normal control (NC), (2) Hyperuricemia group (PO-injected, PO), (3) PO + COE 200 mg/kg (COE200), (4) PO + COE 100 mg/kg (COE100), (5) PO + LO 20 mg/kg (LO20), (6) PO + LO 10 mg/kg (LO10), (7) PO + MO 20 mg/kg (MO20), (8) PO + MO 10 mg/kg (MO10), (9) PO + benzbromarone 50 mg/kg (Benz50). Doses were determined based on preliminary dose–response experiments. COE, LO, and MO were dissolved in 0.5% CMC and administered orally 1 h after PO injection for 5 days.

### 4.7. Collection of Blood, Urine, and Tissue Samples

Blood samples were collected 2 h after the final administration, and serum was separated by centrifugation (2000× *g*, 15 min, 4 °C). Urine samples were collected in metabolic cages 2 h after administration. Serum and urine samples were used for biochemical analyses. Following blood and urine collection, kidney tissues were excised and stored at −80 °C for further assays.

### 4.8. Biochemical Analysis of Serum and Urine

Serum uric acid (SUA) and urinary uric acid (UUA) levels were measured using a commercial uric acid assay kit (Biovision, Milpitas, CA, USA) according to the manufacturer’s instructions. Creatinine (Cr), blood urea nitrogen (BUN), aspartate aminotransferase (AST), alanine aminotransferase (ALT), and lactate dehydrogenase (LDH) levels were analyzed using an automated biochemical analyzer (Hitachi 747, Hitachi, Chiyoda City, Japan). Fractional excretion of uric acid (FEUA) was expressed as: FEUA = [SCr × UUA/SUA × UCr] × 100.

### 4.9. Histopathological Analysis of Kidney Tissues

Kidney tissues were fixed in formalin for 24 h and embedded in paraffin. Tissue sections (7 µm thick) were prepared and stained with hematoxylin and eosin (H&E) for histopathological examination under a light microscope.

### 4.10. Statistical Analysis

Data are presented as mean ± standard error of the mean (SEM). Statistical significance was determined using a one-way analysis of variance (ANOVA) followed by Dunnett’s multiple comparison test using GraphPad Prism 7.0 software. Before conducting ANOVA, we tested for normality using the Shapiro–Wilk test, and tested for homoscedasticity using Bartlett’s test or Brown-Forsythe test. To increase the reliability of the experimental results, the assignment of the experimental and control groups was randomized and the double-blind method was applied when performing statistical processing. A *p*-value < 0.05 was considered statistically significant.

## 5. Conclusions

This study demonstrated that COE effectively inhibits urate uptake through URAT1 modulation, with an IC_50_ value of 3.24 µg/mL. In a hyperuricemia rat model, COE significantly reduced serum uric acid levels and enhanced urinary uric acid excretion. Renal histopathological analysis revealed that COE administration alleviated renal injury, including tubular dilation and inflammatory cell infiltration, while reducing BUN levels, indicating improved renal function. UHPLC-CAD analysis identified MO and LO as the primary active components responsible for the antihyperuricemic effects of COE.

Notably, COE did not induce significant changes in liver function markers (ALT, AST, and LDH), suggesting a favorable safety profile. These findings indicate that COE exerts its antihyperuricemic effects by modulating URAT1 and promoting urate excretion, with potential renoprotective properties. However, further studies are required to elucidate its effects on other urate transporters and to assess its long-term safety and clinical applicability.

## Data Availability

Data will be made available upon request from the corresponding author.

## Supplementary Materials

The following supporting information can be downloaded at: https://www.mdpi.com/article/10.3390/ijms26209980/s1.
