# Supplementary material for: Antihyperuricemic Effects of Cornus officinalis Extract via URAT1 Regulation and Renoprotective Mechanisms
Source: Int J Mol Sci. 2025 Oct 14;26(20):9980. doi: 10.3390/ijms26209980 (PMC12562802; doi:10.3390/ijms26209980)
Supplement: Supplementary file 1 [file ijms-26-09980-s001.zip › ijms-3880146-supplementary.pdf]

Supplementary Materials

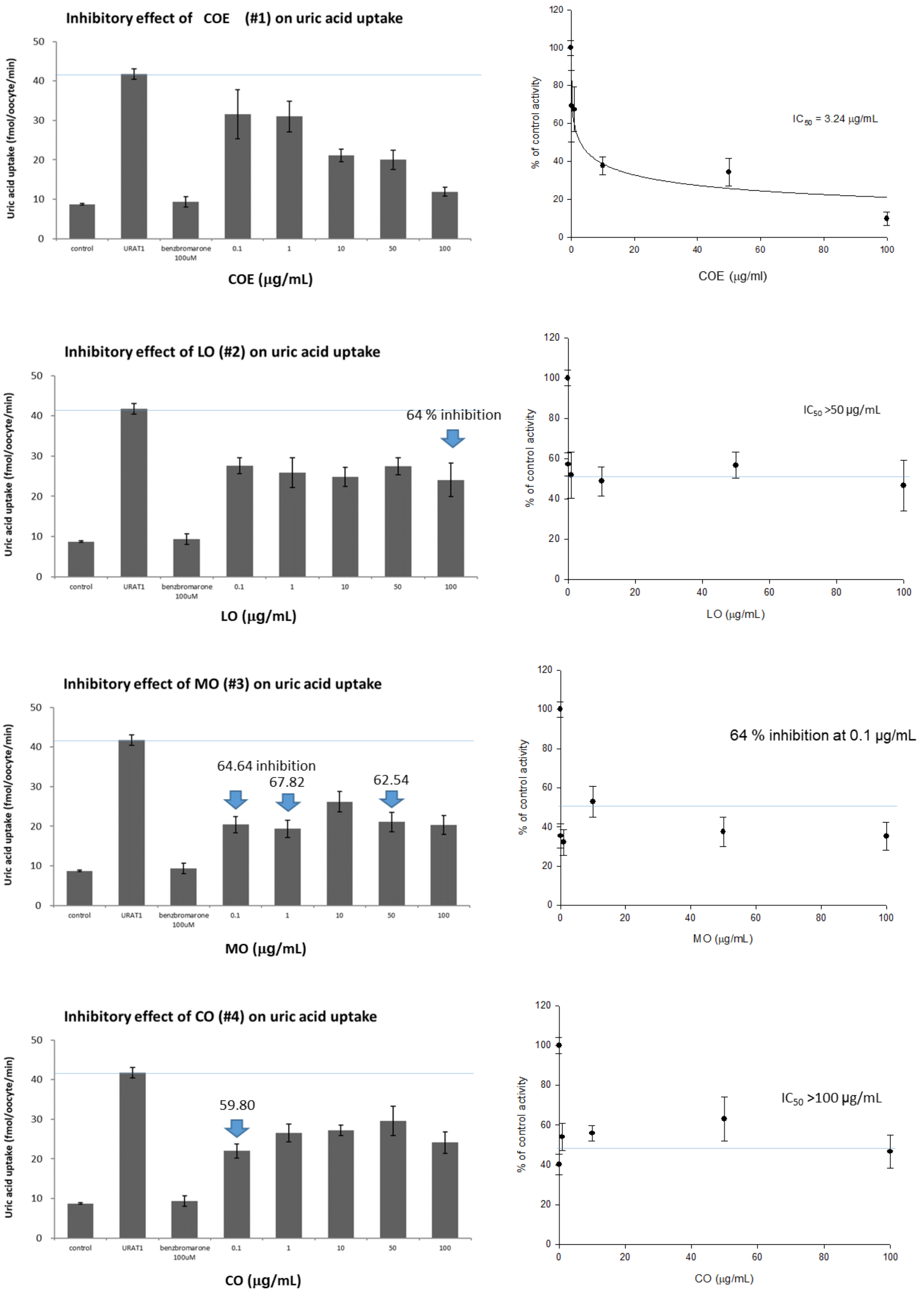

Figure S1. Effects of COE, LO, MO, and CO on urate excretion in *in vitro* URAT1-expressing oocytes (raw uptake rates)

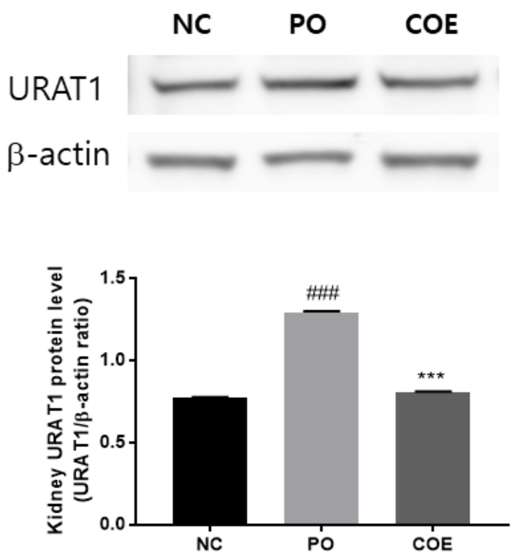

Figure S2. Effects of COE on the expression of URAT1 in the kidney of PO-induced hyperuricemic rats. Renal protein expression levels of URAT1 urate transporter by western blot analysis (n = 5 per group). ###p < 0.05 vs. the NC group; ***p < 0.05 vs. the PO group.

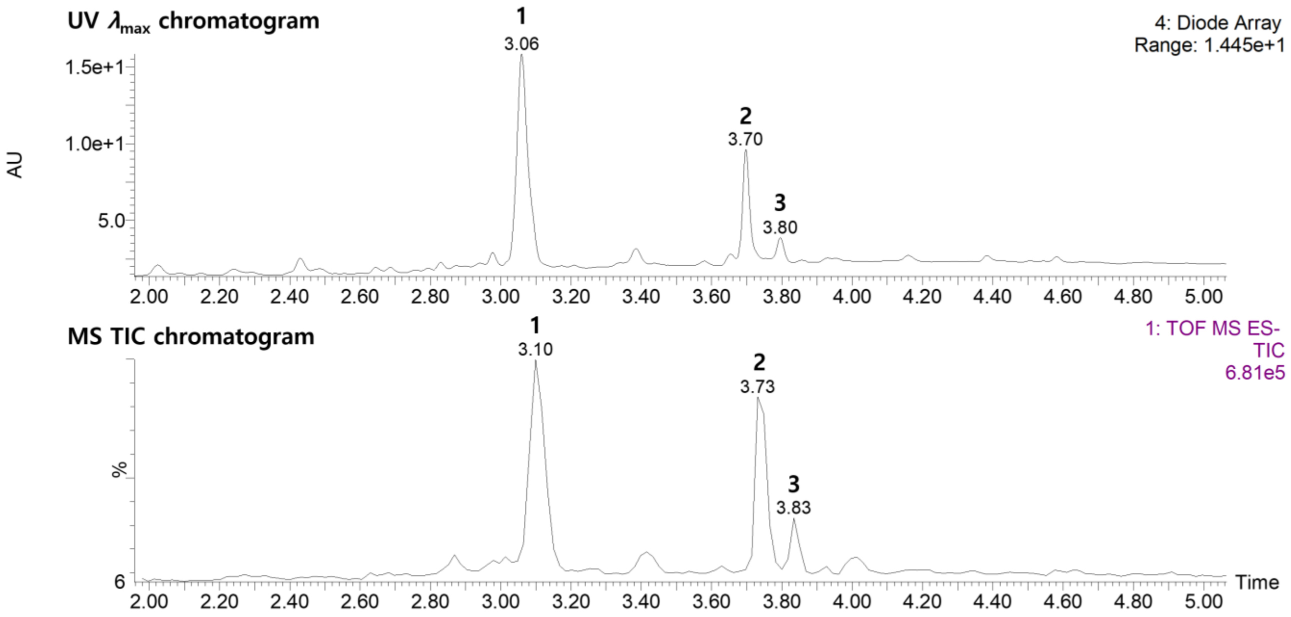

Figure S3. UPLC–DAD and QTof/MS total ion chromatograms of COE. Representative chromatograms showing separation of major constituents. Peaks **1**–**3** correspond to MO, LO, and CO, which were subjected to MS analysis in Figure S4.

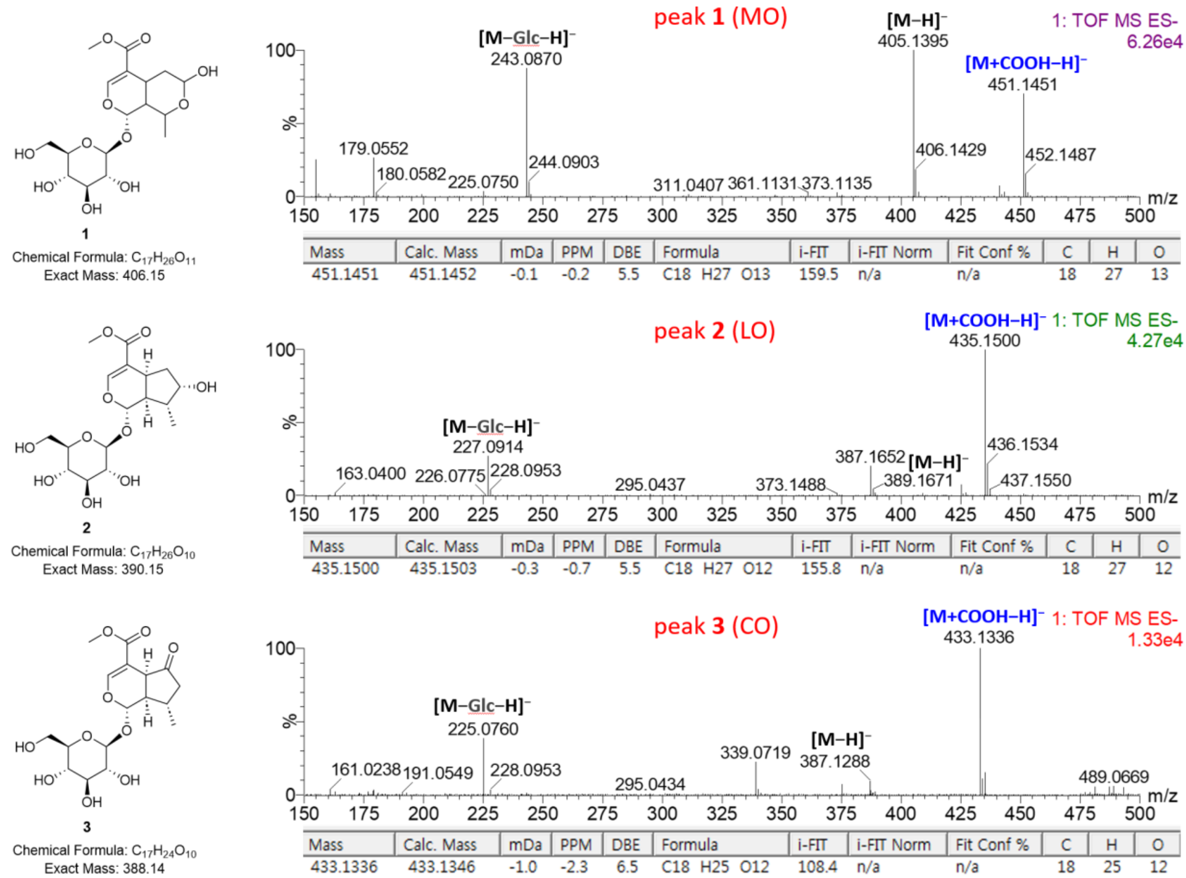

Mass	Calc. Mass	mDa	PPM	DBE	Formula	i-FIT	i-FIT Norm	Fit Conf %	C	H	O
451.1451	451.1452	-0.1	-0.2	5.5	C18 H27 O13	159.5	n/a	n/a	18	27	13

Mass	Calc. Mass	mDa	PPM	DBE	Formula	i-FIT	i-FIT Norm	Fit Conf %	C	H	O
435.1500	435.1503	-0.3	-0.7	5.5	C18 H27 O12	155.8	n/a	n/a	18	27	12

Mass	Calc. Mass	mDa	PPM	DBE	Formula	i-FIT	i-FIT Norm	Fit Conf %	C	H	O
433.1336	433.1346	-1.0	-2.3	6.5	C18 H25 O12	108.4	n/a	n/a	18	25	12

Figure S4. Figure S4. UPLC–QTof/MS spectra of morroniside (peak **1**), loganin (peak **2**), and cornin (peak **3**) from COE. Negative ion mode high-resolution MS spectra and HR-MS reports confirm the identity of the three compounds.

Methods

S1. Western Blot Analysis

Kidney tissues were homogenized in a pro-prep extraction solution (Intron, Seoul, Korea) and then centrifuged (13,000 × g, 4 °C) for 15 min. The supernatant was used for the Western blotting analysis of the targeted proteins. Total protein levels were determined by a DC protein assay. The primary antibodies included β-actin (Santa Cruz, Dallas, TX, USA) and urate transporter 1 (URAT1; MyBioSource, San Diego, CA, USA). The bands from the membrane were visualized by ECL detection reagent using an ImageQuant LAS 4000 (GE Healthcare Life Sciences, Seoul, Korea). The density from the obtained images was determined using Image J1.49 software of NIH (Bethesda, MD, USA). The visualized target protein levels were normalized to β-actin.

S2. UPLC-QTof/MS

UPLC was performed on a Waters Acquity UPLC system coupled with a QTof/mass spectrometer. Aliquots (3.0 µL) of each sample were injected into a BEH C18 column (100 × 2.1 mm, i.d., 1.7 µm) at a flow rate of 0.4 mL/min and eluted using a chromatographic gradient of two mobile phases (A: H_2O containing 0.1% FA; B: ACN containing 0.1% FA). A linear gradient was optimized as follows: 0 min, 10% B; 0–1 min, 10% B; 1–8 min, 10–40% B; 8–11 min, 40–90% B; 11–11.3 min, 90–100% B; 11.3–13.3 min, 100% B; 13.3–13.4 min, 100–10% B; and 13.4–15 min, return to initial elution condition of

10% B. The QTof spectrometer was operated in negative-ion mode in the following conditions: capillary voltage, 2.3 kV; cone voltage, 50 V; source temperature, 110°C; and desolvation temperature, 350°C. A sprayer with a reference solution of leucine-enkephalin ($[M–H]^-$ *m/z* 554.2615) was used as the lock mass. The full scan data and MS/MS spectra were collected using MassLynx 4.1 software (Waters Corporation, Milford, MA, USA).
